# Application of Monoclonal Antibodies against Bioactive Natural Products: Eastern Blotting and Preparation of Knockout Extract

**DOI:** 10.1155/2012/260425

**Published:** 2012-02-14

**Authors:** Hiroyuki Tanaka, Osamu Morinaga, Takuhiro Uto, Shunsuke Fuji, Frederick Asare Aboagye, Nguyen Huu Tung, Xiao Wei Li, Waraporn Putalun, Yukihiro Shoyama

**Affiliations:** ^1^Graduate School of Pharmaceutical Science, Kyushu University, 3-1-1 Maidashi, Higashi-ku, Fukuoka 812-0855, Japan; ^2^Faculty of Pharmaceutical Science, Nagasaki International University, 2827-7 Huis Ten Bosch, Sasebo 859-3298, Japan; ^3^Faculty of Health Management, Nagasaki International University, 2827-7 Huis Ten Bosch, Sasebo 859-3298, Japan; ^4^Phytochemistry Department, Centre for Scientific Research into Plant Medicine, University of Ghana, P.O. Box 73, Mampong-Akuapem, Ghana; ^5^State Key Laboratory of Natural and Biomimetic Drugs, School of Pharmaceutical Sciences, Peking University, No.38 Xue-yuan Road, Haidian District, Beijing 100191, China; ^6^Faculty of Pharmaceutical Science, Khon Kaen University, Khon Kaen 40002, Thailand

## Abstract

Matrix-assisted laser desorption/ionization (MALDI) tof mass spectrometry was used for the confirmation of hapten number in synthesized antigen. As application of MAb, the MAbs against ginsenosides and glycyrrhizin have been prepared resulting in the development of two new techniques that we named the eastern blotting method and the knockout extract preparation. In eastern blotting technique, glycosides like ginsenosides and glycyrrhizin separated by silica gel TLC were blotted to PVDF membrane that was treated with a NaIO_4_ solution followed by BSA resulted in glycoside-BSA conjugate on a PVDF membrane. The blotted spots were stained by MAb. Double staining of eastern blotting for ginsenosides using antiginsenoside Rb_1_ and Rg_1_ MAbs promoted complete identification of ginsenosides in *Panax* species. The immunoaffinity concentration of glycyrrhizin was determined by immunoaffinity column conjugated with antiglycyrrhizin MAb resulting in the glycyrrhizin-knockout extract, which was determined by the synergic effect with glycyrrhizin on NO production using the cell line.

## 1. Introduction

Immunoassay systems using monoclonal antibody (MAb) against drugs and small molecular weight bioactive compounds have become an important tool for studies on receptor binding analysis, enzyme assay, and quantitative and/or qualitative analytical techniques in animals or plants. The immunoblotting method is based on the western blotting technique that utilizes the antigen-antibody binding properties and provides a specific and sensitive detection of higher molecule analytes like peptides and proteins. In our ongoing study on MAb, previously we prepared various kinds of MAb against natural products like forskolin [1], solamargine [2], crocin [3], marihuana compounds [4], opium alkaloids [5], ginsenosides [6, 7], berberine [8], sennosides [9], paeoniflorin [10], glycyrrhizin [11, 12], ginkgolic acid [13], aconitine alkaloid [14], baicalin [15], and so on and developed individual competitive enzyme-linked immunosorbent assay (ELISA) as a highly sensitive, specific, and simple methodology.

The confirmation of hapten number in synthesized antigens is most important in the first stage of MAb preparation. Therefore, its determination method will be discussed first of all. As an application of MAb, the MAb against ginsenosides and glycyrrhizin has been prepared resulting in the development of two new techniques that we have named the eastern blotting method [12] and the knockout extract preparation [16]. They will be introduced in this paper.

## 2. Preparation of MAb against Natural Products

Various methods have been employed for the determination of natural products. They include spectral methods such as infrared (IR), nuclear magnetic resonance (NMR), and circular dichroism (CD) and other chromatographic methods such as ion chromatography (IC), capillary electrophoresis (CE), and high-speed counter current chromatography (HSCCC), and so on. Compared to TLC, GLC and HPLC methods, the ELISA method was more sensitive and selective. Moreover, it is possible to study a large number of natural products. Since natural product extracts consist of various chemical constituents (e.g., licorice contains 470 components or more), in general, some pretreatment is necessary for HPLC and other chromatographic analysis methods. ELISA, however, can determine the concentration of components directly without any pretreatment. Therefore, ELISA was used to measure the concentration of ginsenoside Rb_1 _in ginseng and traditional Chinese medicines (TCMs).

### 2.1. Analytical Method for Determination of Hapten Number in Antigen, Hapten-Carrier Protein Conjugate

For production of MAb, synthesis of hapten, which is derived from an immune antigen and linker bridge, and the carrier protein conjugate is necessary. There had been no direct and appropriate methods for the determination of haptens conjugated with carrier proteins without differential UV analysis, radiochemical, or chemical methods. Therefore, immunization by the injection of hapten-carrier protein conjugate was unreliable. Wengatz et al. [17] determined the hapten density of immunoconjugates by matrix-assisted UV laser desorption/ionization mass spectrometry. We also reported the direct analytical method of hapten and carrier protein conjugates by a matrix-assisted laser desorption/ionization mass spectrometry (MALDI) tof mass spectrometry using an internal standard [18–20].

**Figure 1:**
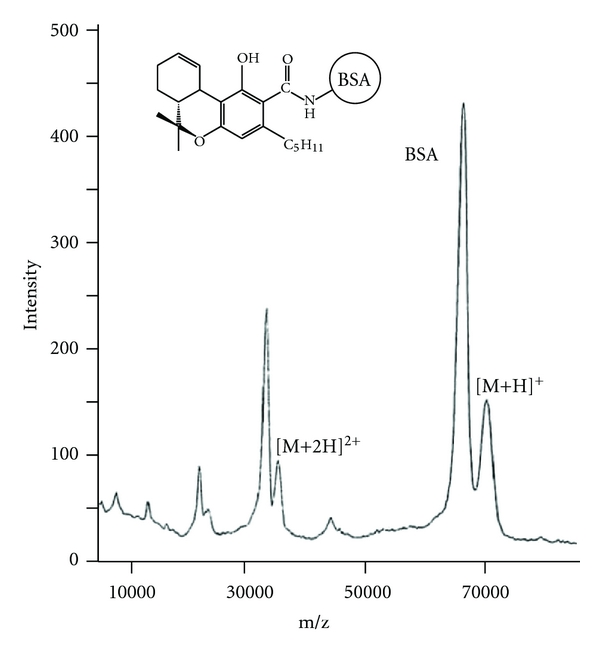
Matrix-assisted laser desorption/ionization tof mass spectrometry of tetrahydrocannabinolic-acid-BSA conjugate. [M + H] indicates the molecular weight of the conjugate, from which the hapten number can be calculated.

Figure 1 shows the MALDI tof mass spectrum of the most pharmacologically active marihuana compound, tetrahydrocannabinolic acid (THCA)-bovine serum albumin (BSA) conjugate, and BSA used as an internal standard [20]. This shows only the singly, doubly, and triply ionized molecule ions of the intact conjugate. The sharp peak at m/z 66,465 is the [M + H]^+^ peak of BSA. A small [M + H]^+^ peak of the THCA-BSA conjugate is at m/z 70,792, indicating that the calculated molecular mass of the THCA-BSA conjugate is 70,581 using a calculated molecular mass of 66,267 for BSA. The calculated molecule mass of the THCA moiety is 4,314. From this result, 12.7 molecules of THCA are combined with BSA [20]. Since this method is suitable for small molecule natural products, we had been analyzing the hapten number of all natural products for MAbs including glycosides like ginsenosides and glycyrrhizin.

### 2.2. Preparation of MAb against Ginsenosides and ELISA as an Assay System

Ginseng, the crude drug of *Panax ginseng*, is one of the most important natural medicines in many countries. It has been used to enhance stamina and capacity to cope with fatigue and physical stress and as a tonic against cancers, disturbances of the central nervous system (memory, learning, and behavior), hypothermia, carbohydrate and lipid metabolism, immune function, the cardiovascular system, and radioprotection [21]. It contains more than 30 kinds of dammarane and oleanane saponins considered to be pharmacologically active components. Ginsenoside Rb_1_ is the main saponin in ginseng. However, since the concentration in the ginseng root or the root extract varies depending on the method of extraction, subsequent treatment, or even the season of its collection [22], standardization of quality is required. For this purpose, we have prepared anti-ginsenoside Rb_1_ [6] and Rg_1_ MAbs [7]. The immunoassay system using MAb is not frequently used for naturally occurring smaller molecular weight bioactive compounds. Preparation of MAbs is difficult, but is one of the most important steps for the analysis of natural products. As a typical natural product, the preparation of MAb against the ginseng saponin ginsenoside Rb_1_ will be discussed.

A hybridoma-producing MAb reactive to ginsenoside Rb_1_ was obtained by the general procedure and classified into IgG2b which had *κ* light chains. The reactivity of IgG type MAb, 9G7 was tested by varying antibody concentration and by performing a dilution curve. The antibody concentration was selected for competitive ELISA. The free MAb following competition is bound to polystyrene microtiter plates precoated with ginsenoside Rb_1_-HSA. Under these conditions, the full measurement range of the assay extends from 20 to 400 ng/mL [6].

Cross-reactivity is the most important factor in determining the value of an antibody. Since the ELISA for ginsenoside Rb_1_ was established for phytochemical investigations involving crude plant extracts, the assay specificity was checked by determining the cross-reactivity of the MAb with various related compounds. The cross-reactivity data of MAb that was obtained were examined by competitive ELISA and calculated using picomole amounts of ginsenoside Rb_1_. The cross-reactivity of ginsenoside Rc and Rd, which possess a diglucose moiety attached to the C-3 hydroxy group, was weak compared to ginsenoside Rb_1_ (0.024 and 0.020%, resp.). Ginsenoside Re and Rg_1_ showed no cross-reactivity (less than 0.005%). It is evident that the MAb reacted only with a small number of structurally related ginsenoside Rb_1_ molecules, and very weakly, and did not react with other steroidal compounds like glycyrrhizin, digitoxin, tigogenin, tigonin, and solamargine.

In our ongoing studies on MAbs against ginseng saponins, anti-ginsenoside Rg_1_ MAb [7] and Re [23] have been prepared and their ELISA was set up. Anti-ginsenoside Rg_1_ MAb was also highly specific like anti-ginsenoside Rb_1_. On the other hand, anti-ginsenoside Re MAb showed wide cross-reactivity. Therefore, the MAb can be used for the analysis for the total ginsenoside concentration.

### 2.3. Application of MAb in the Natural Products Field

Although western blotting is a common assay methodology for high-molecular-weight-substances, this method has not been employed for small molecules, as direct immunostaining of such compounds on a TLC plate is as yet unknown. Therefore, a new method for such small-molecular compounds is required. Moreover, if small molecules can be blotted to a membrane, fixing them also requires a new methodology. Previously, we succeeded in separating small-molecule compounds such as solasodine glycosides into a part of an epitope and fixing on the membrane [24], as follows.

#### 2.3.1. New Staining Method for Ginsenosides, “Eastern Blotting”

**Figure 2:**
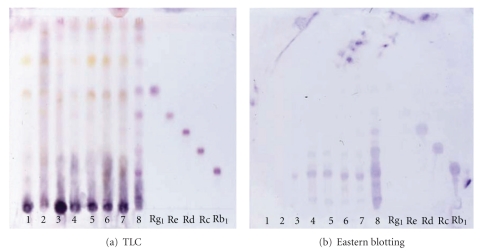
Eastern blotting of ginsenosides in traditional Chinese medicine formulas using anti-ginsenoside Rb1 MAb. Samples (1) Jigengtang, (2) Dahuanggancaotang, (3) Nenshenyangyongtang, (4) Sijunzitang, (5) Nenshentang, (6) Buanxiaxiexintang, (7) Xiaocaihutang, and (8) Crude ginseng extract. Lanes 1 and 2 do not contain ginseng. Standard of ginsenosides indicated, ginsenosidr-Rg_1_, -Re, -Rd, -Rc, and -Rb_1_, respectively, from the upper spot.

Figure 2 shows the H_2_SO_4_ staining and eastern blotting of ginsenoside standards and TCM using anti-ginsenoside Rb_1_ MAb. It is impossible to determine the ginsenosides by TLC staining by H_2_SO_4_ as indicated in Figure 2(a). On the other hand, clear staining of ginsenoside Rb_1_ occurred by eastern blotting, as indicated in Figure 2(b). Furthermore, it became evident that Jigengtang and Dahuanggancaotang prescriptions that did not contain ginseng, as indicated by the absence of a ginsenoside Rb_1 _band. The eastern blotting method was considerably more sensitive than that of H_2_SO_4_ staining. The H_2_SO_4_ staining detected all standard compounds. The eastern blotting indicated only limited staining of ginsenoside Rb_1_, Rc, and Rd, whose cross-reactivities were under 0.02% as shown in Figure 2(b). We suggest that an aglycone, protopanaxadiol, and a part of the sugars may be of importance to the immunization and may function as an epitope for the structure of ginsenosides. In addition, it is suggested that the specific reactivity of sugar moieties in the ginsenoside molecule against anti-ginsenoside Rb_1_ MAb may be modified by the NaIO_4_ treatment of ginsenosides on the PVDF membrane, causing ginsenoside Rc and Rd to become detectable by eastern blotting.

**Figure 3:**
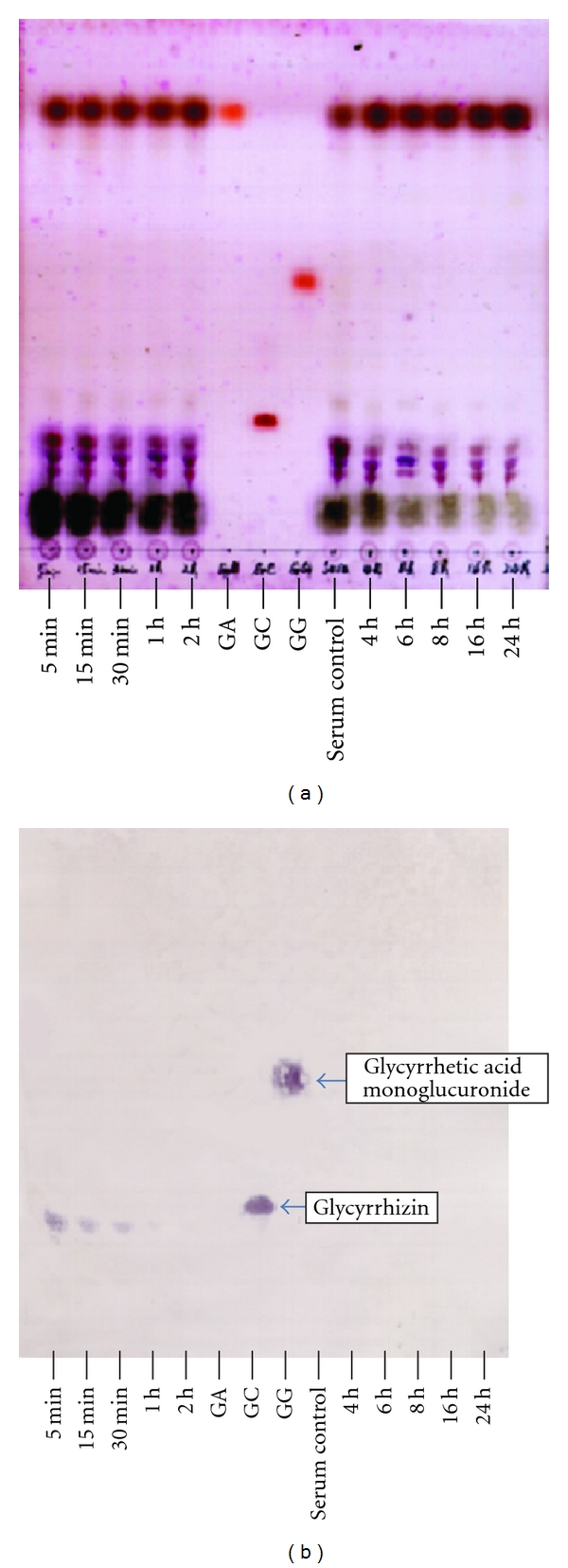
Eastern blotting profile of glycyrrhizin in rat serum after injection of glycyrrhizin (a) TLC of rat-serum-injected glycyrrhizin TLC was developed with *n*-BuOH-H_2_O-AcOH (7 : 2 : 1). Spots were detected by 10% H_2_SO_4_. (b) Eastern blotting of rat-serum-injected glycyrrhizin; the band of glycyrrhizin was detected until 1 h after injection. GA: glycyhrrhetinic acid, GC: glycyrrhizin, and GG: glycyrrhetinic acid monoglucuronide.

Application of the eastern blotting method for the detection of glycyrrhizin in the serum samples was investigated. In general, it is difficult to detect glycyrrhizin in serum due to the large amount of impurities. The developed bands of impurities lapped over the band of glycyrrhizin, and we failed to find a proper developing solvent system that could separate glycyrrhizin and the impurities apart from each other clearly on the TLC plate.  Figure 3 shows the detection of glycyrrhizin by the eastern blotting technique in the rat serum samples. Glycyrrhizin could not be identified on the TLC plate stained by H_2_SO_4 _through many bands that were detected (Figure 3(a)). On the other hand, eastern blotting clearly shows the band of glycyrrhizin even after 1 h (Figure 3(b)). Although the sensitivity of the eastern blotting method was greatly affected by the impurities, the detection limit was still at the nanogram level. The results proved that the eastern blotting technique could be a unique method for identifying glycyrrhizin against a background of a large amount of impurities. 

**Figure 4:**
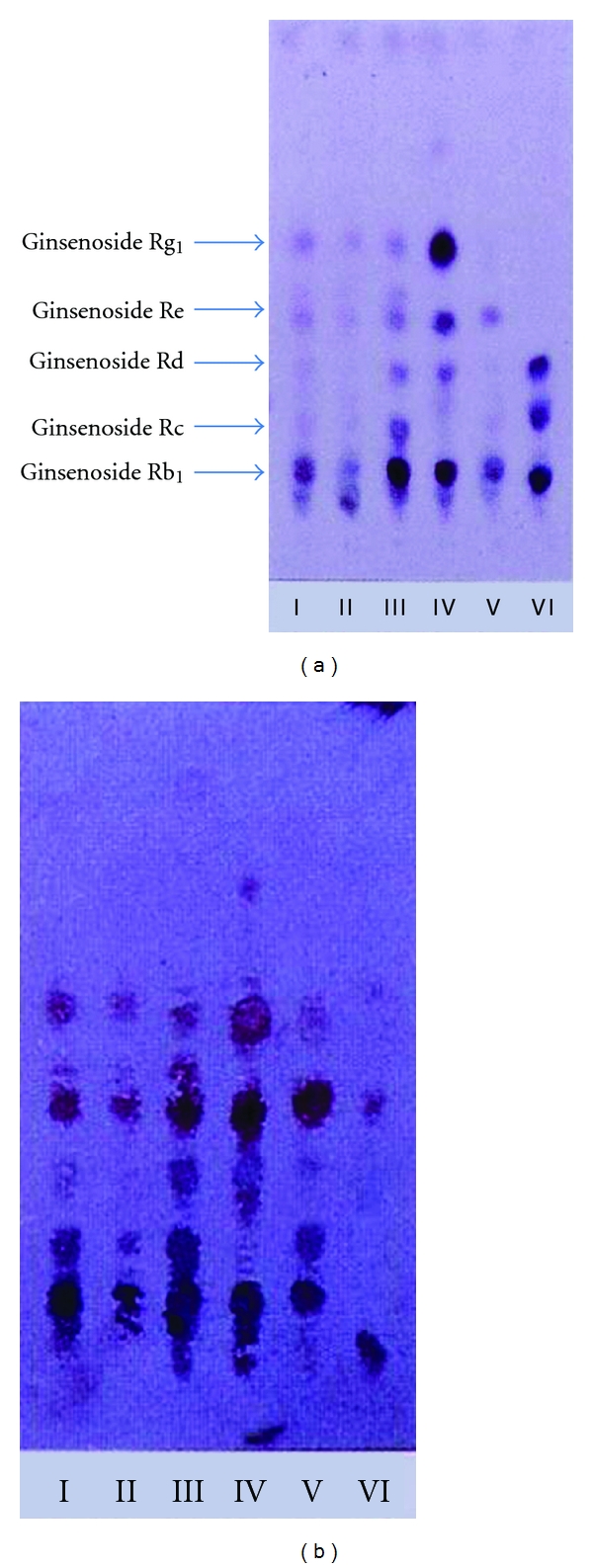
Double Eastern blotting staining of ginsenosides contained in various ginseng samples using anti-ginseenoside-Rb1 and anti-ginsenoside-Rg_1_ MAbs. (a) TLC profile stained by sulfuric acid. (b) Eastern blotting by anti-ginsenoside-Rb_1_ and anti-ginseside-Rg_1_ monoclonal antibodies I, II, III, IV, V, and VI indicated white ginseng, red ginseng, fibrous ginseng (*P. ginseng*), *P*. *notoginseng*, *P*. *quinquefolius* and *P*. *japonicus*, respectively. Upper purple color spots and lower blue color spots were stained by anti-ginsenoside-Rg_1_, and anti-ginsenoside-Rb_1_ monoclonal antibodies, respectively.

When the mixture of anti-ginsenoside Rb_1_ and Rg_1_ MAbs and the pair of substrates were tested for staining for ginsenosides, all ginsenosides, ginsenoside Rb_1_, -Rc, -Rd, -Re, and -Rg_1_ were stained blue although the purple color staining for ginsenoside Rg_1_ was expected because 3-amino-9-ethylcarbazole and 4-chloro-1-naphotol might be different. Therefore, we performed successive staining of the membrane using anti-ginsenoside Rg_1_ and then anti-ginsenoside Rb_1_. Finally, we performed the double staining of ginsenosides indicating that ginsenoside Rg_1_ and ginsenoside Re were stained purple and the other blue, as indicated in Figure 4. From this result, both antibodies can distinguish the individual aglycones, protopanaxatriol, and protopanaxadiol. For this application, the crude extract of various *Panax* species was analyzed by the newly developed double staining system. Major ginsenosides can be determined clearly by the double staining method, as indicated in Figure 4.

Therefore, it is suggested that the staining color shows the pharmacological activity. As shown in Figure 4(b), the purple bands indicate ginsenosides which have protopanaxatriol as an aglycone and stimulation activity for the central nervous system (CNS). On the other hand, the blue color indicates ginsenosides containing protopanaxadiol as an aglycone that possess a depression effect on the CNS. Moreover, the Rf value of ginsenosides roughly suggests the number of sugars attached to the aglycone. Both analyses make it possible to jointly identify which aglycone attaches and how many sugars it possesses, leading to the structure of the ginsenosides. In fact, three kinds of ginsenosides possessing protopanaxadiol-ginsenoside Rh_1_, -Rf, and 20-*O*-glucoginsenoside Rf in *P. ginseng* root were determined by coloring and Rf value by comparing them with the structures reported in the previous paper [25].

**Figure 5:**
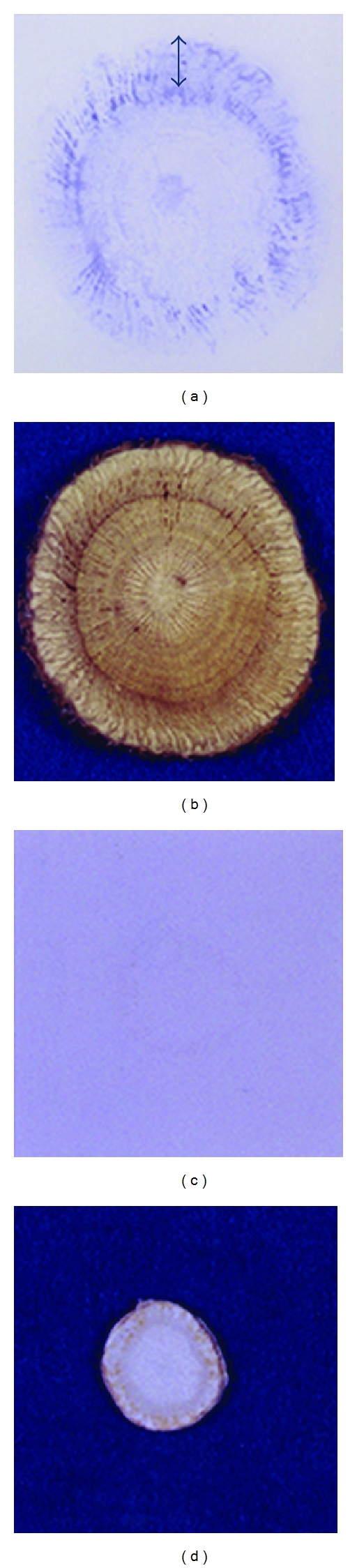
Immunocytolocalization of glycyrrhizin in fresh licorice root using anti-glycyrrhizin MAb. (a) Eastern blotting of fresh licorice slice, (b) fresh licorice slice, (c) Eastern blotting of fresh ginseng slice, and (d) fresh ginseng slice.

Figure 5(a) indicates immunolocalization of glycyrrhizin in a licorice root slice using anti-glycyrrhizin MAb as another application of the eastern blotting method. The phloem (*⟷*) contained a higher concentration of glycyrrhizin than the xylem and cork part [26]. On the other hand, no staining occurred on ginseng (Figure 5(c)).

In the earlier experiments, we carried out the blotted staining on PVDF membrane using MAb on solasodine glycosides and called it western blotting [24]. Now we have applied this new methodology to licorice glycoside, glycyrrhizin and named it eastern blotting [12] for studying ginsenosides [23], saikosaponin [27], and so on.

#### 2.3.2. Immunoaffinity Concentration and One-Step Purification of Ginsenoside Rb_1_ by Immunoaffinity Column

**Figure 6:**
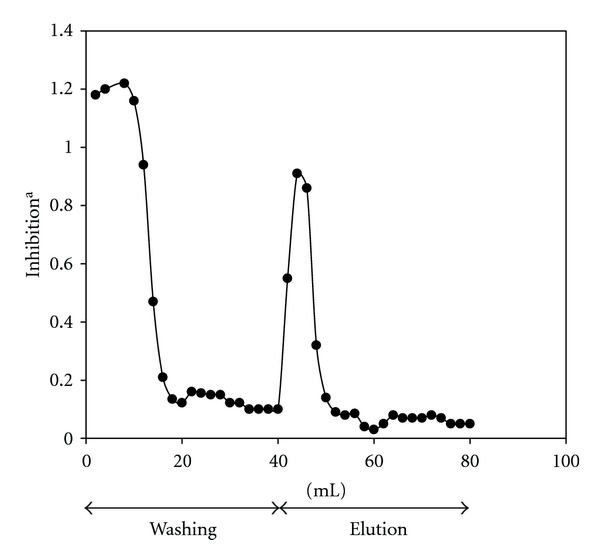
Elution profile of *Panax ginseng* crude extract on immunoaffinity column monitoring by ELISA using anti-ginsenoside-Rb_1_ MAb. The column was washed by phosphate buffer and then eluted by HOAc buffer containing KSCN and MeOH. Individual fractions (2 mL) were assayed by ELISA using anti-ginsenoside-Rb_1_ MAb.

A crude extract of *P. ginseng* roots was loaded onto the immunoaffinity column and washed with the washing solution of phosphate buffer.  Figure 6 shows the fraction 1–8 containing overloaded ginsenoside Rb_1_. The other ginsenosides Rg_1_, Rc, Re. and Rd were also detected in these fractions by eastern blotting (data not shown). A sharp peak appeared around fraction 20–24 eluted by acetate buffer containing KSCN and methanol to give pure ginsenoside Rb_1_.

Overloaded ginsenoside Rb_1_ was repeatedly immunoaffinity column choromatographed to separate ginsenoside Rb_1_ completely [28]. The antibody was stable when exposed to the eluent, and the immunoaffinity column showed almost no decrease in capacity after repeated use more than 10 times under the same conditions, as was reported for a one-step separation of forskolin from a crude extract of *Coleus forskohlii* root [29]. This methodology is effective for the rapid and simple purification of ginsenoside Rb_1_ and may open up a wide field of comparable studies with other families of saponins for which an acceptable method for one-step separation has not yet been developed. Furthermore, to separate the total ginseng saponins, a wide cross-reactive MAb against ginsenoside like anti-ginsenoside Re MAb could be designed, as was done for the total solasodine glycosides by an immunoaffinity column using an anti-solamargine MAb [30]. A combination of immunoaffinity column chromatography, Eastern blotting and ELISA could be used to survey low concentrations of ginsenoside Rb_1_ of plant origin and/or in experimental animals and humans. In fact, we have succeeded in the isolation of ginsenoside Rb_1_ from a different plant, *Kalopanax pictus* Nakai, which was not known previously to contain ginsenosides, using this combination of methods [31].

#### 2.3.3. Newly Established Knockout Extract for Glycyrrhizin

Glycyrrhizin has been reported to possess numerous pharmacological effects like anti-inflammation [32], antiulcer [33], antitumor [34], antiallergy [35], and hepatoprotective activities [36]. Although glycyrrhizin is supposed to be a major active principle in licorice crude extract, a large number of studies have demonstrated that the licorice extract is rich in bioactive compounds other than glycyrrhizin such as triterpenes, flavonoids, and their aglycones [37]. In order to confirm the effect of glycyrrhizin in TCM, previously we purified glycyrrhizin from TCM using an immunoaffinity column conjugated with anti-glycyrrhizin MAb [38]. In this section, one-step purification of glycyrrhizin and its function in the licorice crude extract have been indicated as previously discussed about ginsenoside Rb_1_.

 To eliminate glycyrrhizin from licorice extract, 12 mg of licorice extract (glycyrrhizin content: 1275.0 *μ*g) in loading buffer was applied on the anti-glycyrrhizin MAb immunoaffinity column, and then the loading buffer was continuously circulated through the column to enhance the binding efficiency. After overnight circulation at 4°C, the unbound column fraction was collected. The column was washed, and then bound column fraction was eluted by elution solvent. Glycyrrhizin concentration of bound column fraction was 1269.26 *μ*g of glycyrrhizin (99.5% of the loading glycyrrhizin). On the other hand, glycyrrhizin content of unbound fraction was 3.50 *μ*g (0.27% of the loading glycyrrhizin). These data indicated that anti-glycyrrhizin MAb immunoaffinity column could eliminate glycyrrhizin from licorice crude extract with high efficiency. From this evidence, we named this fraction knockout extract [16, 39].

**Figure 7:**
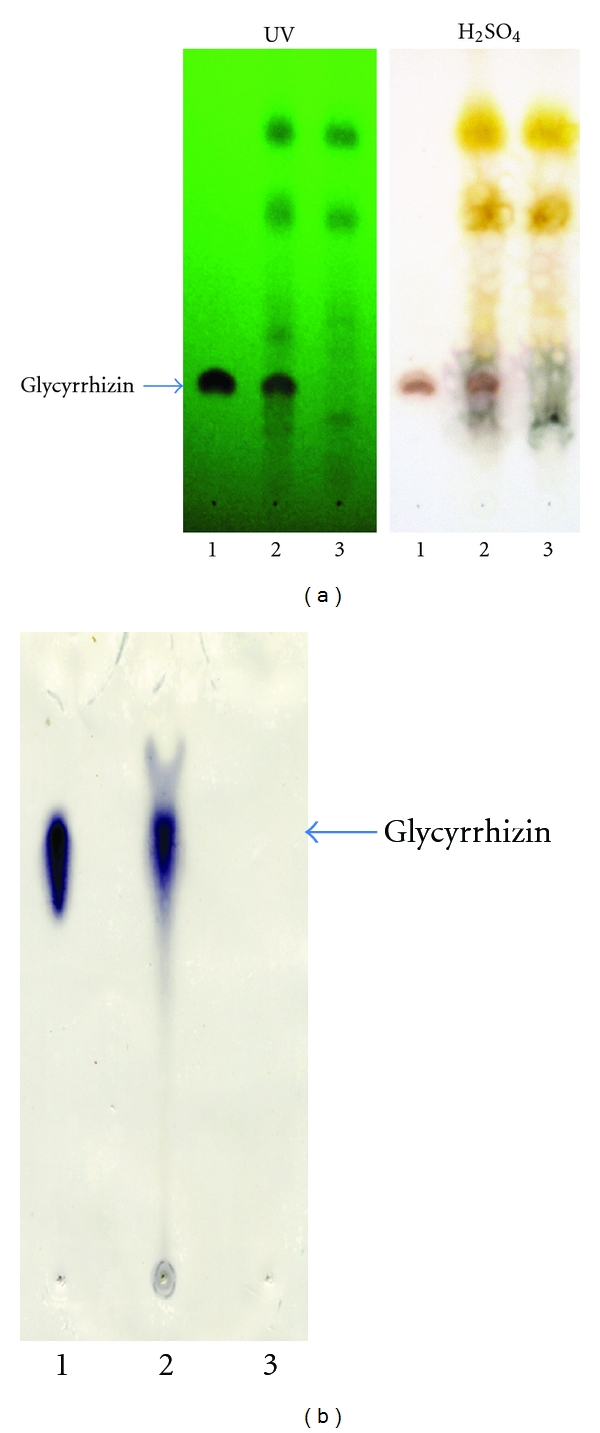
Preparation of knock-out extract eliminated glycyrrhizin from licorice crude extract using immunoaffinity column conjugated with anti-glycyrrhizin MAb. Lines 1, 2, and 3 indicate glycyrrhizin, crude extract, and knock-out extract, respectively.

To further characterize glycyrrhizin knockout extract, the TLC analysis and eastern blotting were performed. As shown in Figure 7(a), several spots including glycyrrhizin were detected in licorice extract. However, the spots of glycyrrhizin were undetected in glycyrrhizin-knockout extract, although all other spots were clearly detected (Figure 7(a), lane 2). eastern blotting by anti-glycyrrhizin MAb demonstrated that glycyrrhizin was detected in licorice extract, but the spot of glycyrrhizin was invisible in unbound fraction (Figure 7(b), lane 2). Therefore, these data suggest that glycyrrhizin was specifically eliminated from licorice extract by anti-glycyrrhizin MAb binding immunoaffinity column.

This technique is useful approach to clarify the multicomponent interaction with the specific protein such as enzymes, but it is unsuitable to investigate the interaction between one principal component and other components in herbal medicines. To investigate the role of one principal compound in the crude extract, we prepared knockout extract by removing target compound from the crude extract using high-specific MAb immunoaffinity column. By using this approach, it may become possible to determine the potential function of one natural compound on the crude extract or TCM by *in vitro* and *in vivo *assays.

Nitric oxide (NO) is free radical with multiple physiological functions, such as vasodilatation, neurotransmission, and inflammation [40]. During inflammatory process, large amount of NO is produced by inducible nitric oxide synthase (iNOS) by inflammatory cytokines and/or bacterial lipopolysaccharide (LPS) in various cell types including macrophages [41]. Overproduction of NO by iNOS triggers the pathogenesis of septic shock and organ destruction in certain inflammatory and autoimmune diseases [42–44]. Therefore, the inhibition of NO production by blocking iNOS expression may be useful strategy for the treatment of various inflammatory diseases.

To determine whether licorice crude extract inhibits NO production in LPS-stimulated mouse RAW264 macrophages, cells were treated with various concentrations of licorice extract for 30 min and then stimulated with LPS. LPS caused a dramatic increase in NO production, and this induction was inhibited in a dose-dependent manner by treatment with licorice extract.

**Figure 8:**
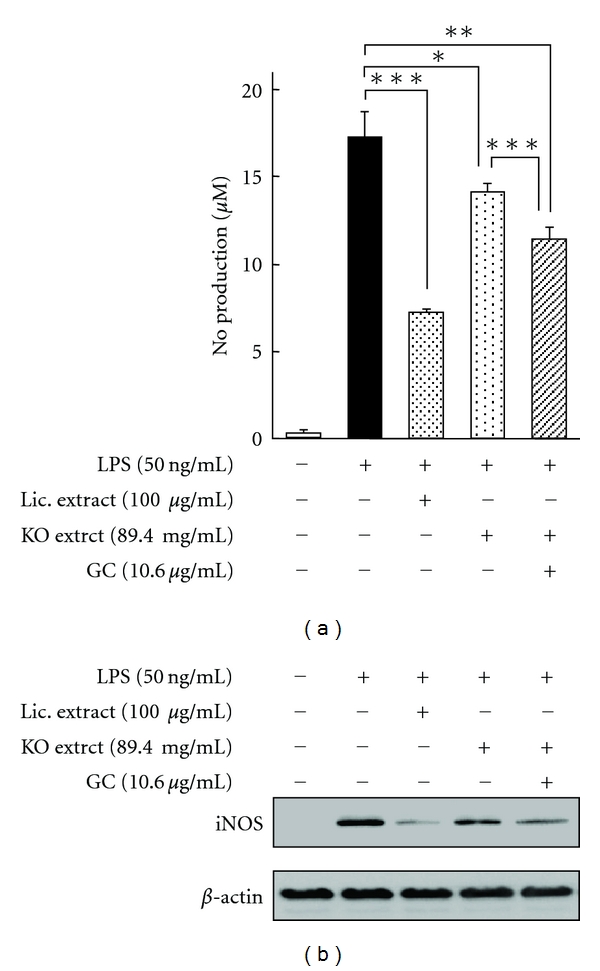
Effects of glycyrrhizin-knockout extract and the combination of glycyrrhizin-knockout extract and glycyrrhizin on LPS-induced NO production (a) and iNOS protein expression (b). *P < 0.05, **P < 0.01, ***P < 0.001.

We next investigated the inhibitory effect of glycyrrhizin-knockout extract and the combined treatment with glycyrrhizin-knockout extract and glycyrrhizin on NO production. Since 100 *μ*g/mL of licorice extract contains 10.6 *μ*g of glycyrrhizin, the cells were pretreated with licorice extract (100 *μ*g/mL), glycyrrhizin-knockout extract (89.4 *μ*g/mL), or combination of glycyrrhizin-knockout extract (89.4 *μ*g/mL) and glycyrrhizin (10.6 *μ*g/mL).  Figure 8(a) indicated that treatment of licorice extract leads to a marked suppression of NO production as compared to LPS treatment [inhibition ratio (IR) = 57.7%]. Interestingly, although glycyrrhizin alone could not block NO production, the inhibitory effect of glycyrrhizin-knockout extract was decreased compared with licorice extract (IR = 17.8%). Moreover, the combined treatment with glycyrrhizin-knockout extract and glycyrrhizin significantly improved the inhibition of NO production (IR = 33.5%). To determine whether the combinational effect of glycyrrhizin-knockout extract and glycyrrhizin was related to iNOS expression, we performed western blotting (Figure 8(b)). Although the licorice extract strongly suppressed LPS-induced iNOS expression, treatment of glycyrrhizin-knockout extract reduced this effect [45].

## 3. Conclusions

Two unique applications using MAb, eastern blotting and knockout extract have been introduced in this paper. The eastern blotting method has great potential applications for the wide range of natural products, especially glycosides like ginsenosides of ginseng and glycyrrhizin in licorice. When two kinds of MAbs, against ginsenoside Rb_1_ and Rg_1_ can be used, the double staining that enhanced the separate staining of ginsenosides having protopanaxatriol or protopanaxadiol in a molecule, occurred. The staining color can be used to monitor the pharmacological activity suggesting that the purple spots contain protopanaxatriol as an aglycone indicating central nervous system (CNS) stimulatory activity [46]. On the other hand, the blue color indicates ginsenosides having protopanaxadiol which possess a depression effect on the CNS [37]. Furthermore, the Rf value of ginsenosides suggests the number of sugars attached to the aglycone. Both evidences make it possible to confirm which aglycone is attached and how many sugars are combined with the aglycone.

We demonstrated that knockout extract prepared by anti-natural compound-specific MAb immunoaffinity column is a useful approach for determination of potential function of natural compound on *in vitro* and *in vivo *assays. The pharmacological analysis by knockout extract might be directly applicable to both other crude extracts and various TCMs to clarify the real pharmacologically active components.

A ginsenoside Rb_1_ knockout extract can be prepared using an immunoaffinity column conjugated with anti-ginsenoside Rb_1_ MAb. In this extract, all compounds except only ginsenoside Rb_1_ are contained. Furthermore, glycyrrhizin-knockout extract prepared from licorice crude extract using an immunoaffinity column conjugated with anti-glycyrrhizin MAb. These knockout extracts may be able to support the pharmacological investigation for finding out a really active component in a crude drug and/or TCM. In fact, addition of glycyrrhizin to glycyrrhizin-knockout extract could improve the inhibition of iNOS expression resulting that glycyrrhizin may exert combinational inhibition of iNOS expression when coexisting with the other constituents contained in licorice extract.
